# Supplementary material for: Recent Updates on ALMT Transporters’ Physiology, Regulation, and Molecular Evolution in Plants
Source: Plants (Basel). 2023 Sep 4;12(17):3167. doi: 10.3390/plants12173167 (PMC10490231; doi:10.3390/plants12173167)
Supplement: Supplementary file 1 [file plants-12-03167-s001.zip › Figure S2_Tree_var3_3.pdf]

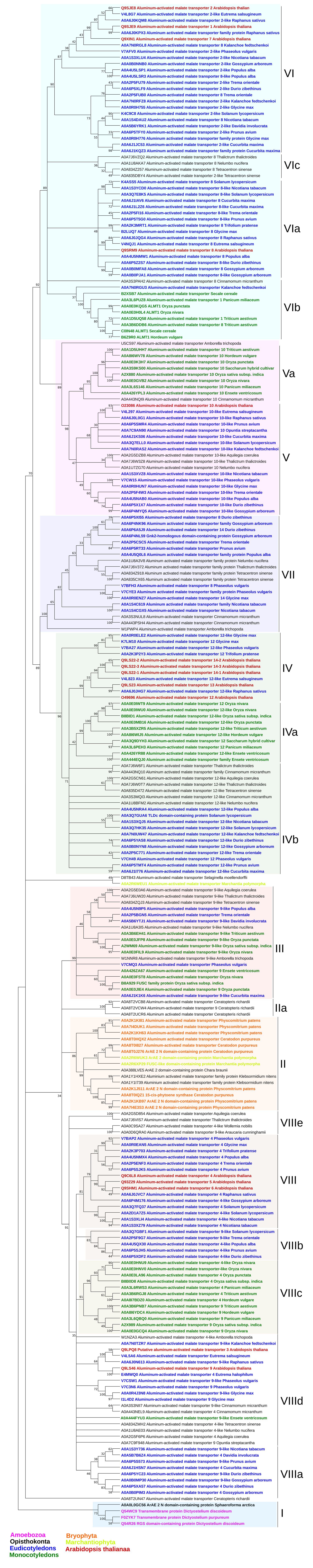
VI
VIc
VIa
VIb
Va
V
VII
IV
IVa
IVb
III
IIa
II
VIIIe
VIII
VIIIb
VIIIc
VIIId
VIIIa
I
Amoebozoa
Opisthokonta
Eudicotyledons
Monocotyledons
Bryophyta
Marchantiophyta
Arabidopsis thalianaa
