# Supplementary material for: Recent Updates on ALMT Transporters’ Physiology, Regulation, and Molecular Evolution in Plants
Source: Plants (Basel). 2023 Sep 4;12(17):3167. doi: 10.3390/plants12173167 (PMC10490231; doi:10.3390/plants12173167)
Supplement: Supplementary file 1 [file plants-12-03167-s001.zip › Supplementary_Table_S1.pdf]

Supplementary Table S1. List of transporters co-localised, co-expressed and/or share domains with ALMTs.

Gene ID	Description
At3g47740	ABC2 homolog 2 (ABCA3)
At5g06530	ABC-2 type transporter family protein (ABCG22)
At3g52310	ABC transporter G family member 27
At3g28960	Transmembrane amino acid transporter family protein
At5g59520	ZRT/IRT-like protein 2(ZIP2)
At2g34960	cationic amino acid transporter 5 (CAT5)
At1g61560	Seven transmembrane MLO family protein (MLO6)
At2g21080	Ras guanine nucleotide exchange factor K
At2g30300	Major facilitator superfamily protein
At3g45710	Major facilitator superfamily protein
At3g23430	putative inorganic phosphate efflux mediator (AtPHO1)
At5g43350	phosphate transporter 1;1 (PHT1;1)
At1g62280	SLAC1 homologue 1 (SLAH1)
At5g24030	putative subunit of SLAH3-SLAH1 anion channel complex (AtSLAH3)
At4g23700	cation/H+ exchanger 17 (CHX17)
At5g38030	MATE efflux family protein
At2g17000	Mechanosensitive ion channel family protein
At5g14940	putative NRT1/PTR subfamily NPF5 transporter (AtNPF5.8)
At4g08300	putative MtN21/SIAR/UmamiT-type amino acid transporter (AtUmamiT17)
At3g02850	putative subunit of Plant-VG voltage-gated potassium cation channel (AtSKOR)
At5g23660	sucrose efflux transporter (AtSWEET12)
At5g14880	putative HAK/KUP/KT cluster-II potassium cation transporter (AtHAK8)
At5g50610	uncharacterized protein
At4g18540	uncharacterized protein
AT5G43180	transmembrane protein of unknown function, DUF599
At2g28780	putative membrane protein of unknown function
At3g09450	putative membrane protein of unknown function
