# Supplementary material for: Recent Updates on ALMT Transporters’ Physiology, Regulation, and Molecular Evolution in Plants
Source: Plants (Basel). 2023 Sep 4;12(17):3167. doi: 10.3390/plants12173167 (PMC10490231; doi:10.3390/plants12173167)
Supplement: Supplementary file 1 [file Figure S1_Multiple_alignment.pdf › Figure S1_Multiple_alignment.pdf]

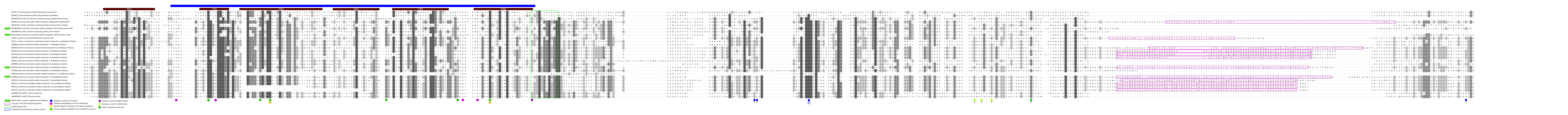
Where ArAE_1 domain signature was identified
The gap in the ALMT domain sequence
GABA-binding motif
Glutamate (E) Essential for Channel Activity
Residues crucial for Al sensing
Residues participating in the Al coordination
Arg (R) residues involved in the malate recognition
The key residues identified in soy GmALMT12 channel
Residue crucial for GABA-binding
Residues crucial for CaM-binding
MAPK S phosphorylation site
